# Identification of IgE and IgG1 specific antigens in *Echinococcus granulosus* cyst fluid

**DOI:** 10.1590/1414-431X20176071

**Published:** 2017-07-03

**Authors:** S. Li, R. Qian, S. Wang, J. Ye, H. Zheng

**Affiliations:** 1 Department of Anesthesiology, First Affiliated Hospital of Xinjiang Medical University, Urumqi, China; 2 Department of Anesthesiology, General Hospital, Xinjiang Command PLA, Urumqi, China; 3 Department of Immunology, Basic Medical College of Xinjiang Medical University, Urumqi, China

**Keywords:** Cystic echinococcosis, Anaphylactic shock, Specific antigens, Specific antibody

## Abstract

Cystic echinococcosis (CE) is an anthropozoonotic disease with worldwide distribution and is caused by the cestode *Echinococcus granulosus*. Anaphylactic shock induced by CE rupture is a serious complication especially in patients with hydatid infections, as the resulting leakage of fluid contains highly toxic endogenous antigen. We aimed to isolate and identify the antigens of specific IgE and IgG1 (sIgE and sIgG1) in *E. granulosus* cyst fluid (EgCF). Crude antigen for EgCF was prepared from *E. granulosus*-infected sheep liver. Antigens were separated and identified by one-dimensional sodium dodecyl sulfate-polyacrylamide gel electrophoresis (1D SDS-PAGE), two-dimensional gel electrophoresis (2-DE), and immunoblotting. Results of 1D SDS-PAGE and immunoblotting showed that 40.5 kDa protein was the major antigen of sIgE, and 35.5 kDa protein was the major antigen of sIgG1 in EgCF. Results of 2-DE and immunoblotting showed that main antigens of sIgE in EgCF were four proteins with pI values ranging from 6.5 to 9.0 and a molecular weight of 40.5 kDa. Main antigens of sIgG1 in EgCF were five proteins with pI values ranging from 6.5 to 9.0 and a molecular weight of 35.5 kDa. The antigens identified for sIgE and sIgG1 can provide critical insights into cellular and molecular mechanisms underlying anaphylactic shock induced by CE.

## Introduction

Cystic echinococcosis (CE) is a worldwide disease caused by the cestode *Echinococcus granulosus*. The prevalence of this disease in epidemic areas is estimated to be 1–7% (1), and the incidence rate is as high as 9% in some localities in Xinjiang, China (2). The overt or unapparent rupture of the parasitic cyst can cause anaphylactic shock, becoming a serious problem (3-5). Patients that develop type I hypersensitivity followed by echinococcosis-induced anaphylactic shock have specific clinical manifestations and immunological characteristics, and usually have poor responses to treatment and poor prognosis (6).

As the antigen components of the *E. granulosus* cyst fluid (EgCF) are complex, researchers have been unable to identify the allergen responsible for the anaphylactic shock; hence, it is difficult to treat the complications effectively. Experiments on animals found that in addition to immunoglobulin E (IgE), IgG1 antibody is also involved in type I hypersensitivity (7,8). IgE-mast cells-histamine pathway has long been associated with anaphylaxis, but an alternative pathway mediated by IgG1 has been suggested to be more important in the elicitation of anaphylaxis (9). Early research suggests that, besides specific IgE (sIgE), anaphylactic shock and even death induced by CE are largely mediated by sIgG, especially sIgG1 subclass (10,11). However, sIgE and sIgG1 antigens produced in EgCF are still not clearly known. Determining the levels of sIgE and sIgG1 in EgCF can be of great significance for assessing the risk of anaphylactic shock, and will certainly provide critical insights into cellular and molecular mechanisms underlying anaphylactic shock induced by CE.

In this study, we conducted experiments to identify the main antigens of sIgE and sIgG1 in EgCF, and to better understand cellular and molecular mechanisms underlying anaphylactic shock induced by CE

## Material and Methods

### Patients and disease controls

The study protocol was approved by the Ethics Committee of the First Affiliated Hospital of Xinjiang Medical University. Patients were mainly from the Altay, Ili Kazakh, Aksu, and Tacheng epidemic areas, and were diagnosed with echinococcosis by surgery at the institution. All patients and healthy volunteers provided written informed consent. In total, 20 patients (T1-T20) admitted for surgery and 10 healthy volunteers (C1-C10) who served as disease controls recruited outside the epidemic area in the Urumqi General Hospital, Lanzhou Command, PLA, China, were enrolled into this study. The diagnosis of anaphylactic shock and classification standard for cysts were the same as used in previous studies (6,11). Serum obtained from patients and healthy volunteers was repackaged and stored at -80°C until further use.

### Inclusion and exclusion criteria

#### Inclusion criteria

Cystic echinococcosis surgical patients who provided written informed consent.

#### Exclusion criteria

Patients who had other infectious diseases at the same time (such as bacterial liver abscess, or hepatitis); and patients who had underlying immune system disease.

#### Healthy volunteer exclusion criteria

Patients with infectious diseases (all kinds of hepatitis); with accompanied immune system disease; and with respiratory infections.

### Preparation of crude antigen of EgCF

EgCF was obtained from infected sheep liver from the local slaughterhouses in Urumqi, China. Freshly isolated EgCF was stored in sterile containers and precipitated for 30 min to remove protoscoleces of *E. granulosus* and then centrifuged (15,000 *g* for 30 min at room temperature). The supernatant used was 3 kDa, obtained by centrifugal ultrafiltration (12,000 *g* for 20 min at room temperature) to obtain the crude antigen of EgCF. The quantity of crude antigen protein concentration used was 12.5 mg/mL, which was repackaged and stored at -80°C until use.

### Screening of serum-positive sIgE and sIgG1 through ELISA

The ELISA experiment followed the method adopted by Liu et al. (12). Ninety-six well plates were coated with 120 μL of 10 μg/mL crude antigen of EgCF. Determination of sIgE concentration was done with patient sera (undiluted) with horseradish peroxidase (HRP)-labeled mouse anti-human IgE antibody added (Southern Biotech, USA; Lot No.: J681-RB83L 1:1000 dilution). Determination of concentration of sIgG1 was done with patient sera (1:10 dilution), with HRP-labeled mouse anti-human IgG1 antibody (SouthernBiotech; Lot No.: L471-NB842 1:1000 dilution) added.

Serum from healthy volunteers was used as control. Absorbance was read at 450 nm on a microplate reader (xMark™ Microplate Spectrophotometer; Bio-Rad, USA). All ELISA experiments were performed in duplicate, and the data obtained are reported as means±SD. Mean absorbance + three standard deviations from controls were used to establish a cutoff value. Patient values greater than the cutoff value were considered to be anti-EgCF sIgE or sIgG1 positive, and serum-positive specimens were used for further immunoblotting experiments.

### One-dimensional SDS-PAGE and IgE and IgG1 immunoblotting

The basic experimental method used was described by Zheng et al. (10). Standard molecular protein markers were provided by Bio-Rad (range 10-250 kDa, Lot No. 161-0374) and Thermo Company (range 10-170 kDa, Lot No. 00102717, USA).

Electrophoresis was performed using a Mini-Protein 3 Cell (Bio-Rad), and the separated proteins were electrotransferred from gel to a nitrocellulose membrane (Hybond-C Extra RPN303E; Amersham Biosciences, Sweden) using Trans-Blot Electrophoretic Transfer Cell (Bio-Rad). The concentrated EgCF proteins were separated using 10% SDS-PAGE.

#### IgE immunoblotting

Patient sera (1:5 dilution) was added with HRP-labeled mouse anti-human IgE antibody (1:3000 dilution). Blots were developed using SuperSignal West Femto Maximum Sensitivity Substrate (Lot no: 34095 Thermo USA), and luminescence was detected on an X-ray film.

#### IgG1 immunoblotting

Patient sera (1:20 dilution) was added with HRP-labeled mouse anti-human IgG1 antibody (1:3000 dilution). Blots were visualized after staining with diaminobenzidine (DAB).

The results of immunoblotting were analyzed using Molecular Imager¯ Gel Doc™ XR+ Imaging System (Bio-Rad) with Image Lab 4.0.1 software.

### 2-DE and immunoblotting

The basic experimental method used was described by Liu et al. (12). To perform two-dimensional gel electrophoresis (2-DE) experiments combined with immunoblotting, 200 μg of crude antigen of EgCF was diluted in 120 μL of IPG (immobilized pH gradient) rehydration buffer. Samples were actively rehydrated and put onto 7-cm pH 3-10 IPG strips (Amersham Biosciences) using Protean IEF cell (Bio-Rad) isoelectric focusing and then separated using SDS-PAGE. 2-DE was performed in triplicate for crude antigen of EgCF under the same conditions for IgE immunoblotting, IgG1 immunoblotting, and Coomassie blue G-250 staining. Sera of T1 patient, in whom sIgE and sIgG1 were all positive and typical anaphylactic shock occurred due to the rupture of parasitic cyst during the surgery, was used. The method of 2-DE IgE immunoblotting experiment was the same as one-dimensional sodium dodecyl sulfate-polyacrylamide gel electrophoresis (1D SDS-PAGE) IgE immunoblotting.

#### IgG1 immunoblotting

Patient sera (1:20 dilution) was added with HRP-labeled mouse anti-human IgG1 antibody (1:3000 dilution). Blots were developed using SuperSignal WestPico (Thermo, Lot no: NCI508), and luminescence was detected on an X-ray film. For a clearer separation of crude EgCF antigen on the 24-cm strip, 2-DE electrophoresis experiments were performed again, similar to the steps for the 7-cm 2-DE. For the preparation of crude antigen of EgCF labeled with Cy3 (Cy3 DIGE kit; GE, Co., Ltd., USA), 800 μg of crude antigen of EgCF was diluted in 450 μL of IPG rehydration buffer. Samples were actively rehydrated into 24-cm pH 3-10 IPG strips (Amersham Biosciences) using Protean IEF cell (Bio-Rad) isoelectric focusing and then separated using SDS-PAGE. Image of the EgCF separated proteins was obtained by fluorescence scanning using a laser scanner.

### Statistical analysis

Statistical analysis was performed using SPSS 15.0 software (SPSS Inc., USA). Absorbance values of the healthy volunteers and patients were compared with single factor analysis of variance (ANOVA).

## Results

The absorbance values in patients and disease controls are reported in Table 1. The cutoff values of IgE and IgG1 were 0.64 and 2.15, respectively. Seven patients with higher IgE and eight patients with higher IgG1 absorbance values than the cutoff were considered to be positive and were chosen for recognition of EgCF antigens of sIgE and sIgG1 using immunoblotting. In patients T1, T10, and T18, anaphylactic shock occurred during surgery and their sera were positive for sIgE and sIgG1.

**Table 1.** Demographic, clinical, and antibody characteristics of patients and disease controls.

Patient	Gender	Age (years)	Location of cysts	Type of cysts	Anaphylactic shock	sIgE Abs value	sIgG1 Abs value
T1	M	35	Liver	Type II	Yes	0.92	2.64
T2	F	36	Liver, spleen	Type II	No	0.50	1.95
T3	M	36	Liver	Type II	No	0.49	1.30
T4	M	33	Liver	Type I	No	0.62	1.57
T5	M	42	Liver	Type II	No	0.64	2.57
T6	F	40	Liver	Type II	No	0.57	2.11
T7	M	25	Liver	Type II	No	1.22	1.90
T8	M	7	Liver	Type II	No	1.58	1.61
T9	F	16	Liver	Type II	No	0.46	2.64
T10	M	42	Liver, lung, pelvic cavity	Type II, Pulmonary	Yes	0.94	2.48
T11	F	51	Liver	Type II	No	0.19	1.45
T12	M	33	Liver	Type II	No	0.33	2.11
T13	M	21	Liver	Type I	No	1.01	2.12
T14	F	60	Liver	Type II	No	0.42	1.09
T15	F	41	Liver	Type II	No	0.93	2.40
T16	F	60	Liver, pelvic cavity	Type II	No	0.61	2.43
T17	M	31	Liver	Type II	No	0.53	2.55
T18	F	10	Lung	Pulmonary	Yes	1.66	2.35
T19	F	46	Liver	Type II	No	0.43	2.13
T20	F	39	Lung	Pulmonary	No	0.63	1.36
C1	M	27				0.41	1.15
C2	F	34				0.52	1.53
C3	M	28				0.43	0.64
C4	F	30				0.36	0.41
C5	M	41				0.35	1.08
C6	F	26				0.42	0.90
C7	M	28				0.44	0.22
C8	F	32				0.55	0.20
C9	M	35				0.42	0.41
C10	F	28				0.30	1.16

T1-T20: patient group; C1-C10: disease control group; M: male; F: female; Yes: anaphylactic shock occurred during surgery; No: anaphylactic shock did not occur during surgery. Abs: absorbance; sIgE: specific immunoglobulin E; sIgG1: specific immunoglobulin G1. Cystic echinococcosis (CE) in hepatic tissues was classified according to ultrasonographic classification. Type I: simple CE: a) overall echo-free; b) with fine echoes. Type II: multiple CE: a) multiple contiguous cysts; b) multi-septated with rosette, honey-comb, or wheel-like pattern. Pulmonary echinococcosis does not have its own classification.

### 1D SDS-PAGE and immunoblotting

**Figure 1.**
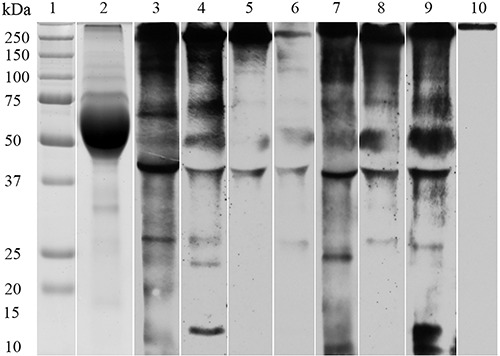
SDS-PAGE and IgE immunoblotting of the crude antigen of *E. granulosus* cyst fluid (EgCF). Crude antigen of EgCF protein extracts were incubated individually with 7 sIgE positive sera (*lanes 3-9*), and with disease control (*lane 10*); crude antigen of EgCF proteins visualized using Coomassie blue R-250 (*lane 2*); protein molecular weight marker (*lane 1*). IgE, immunoglobulin E; SDS-PAGE, sodium dodecyl sulfate-polyacrylamide gel electrophoresis; sIgE, specific immunoglobulin E.

**Figure 2.**
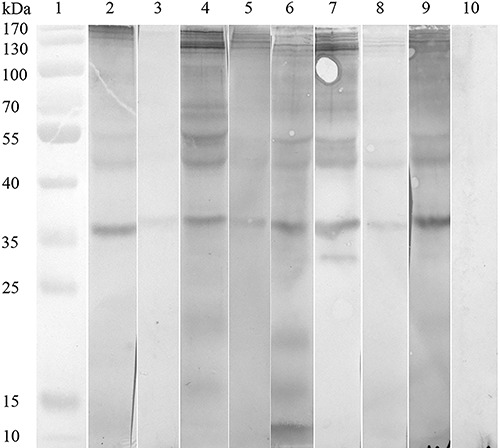
SDS-PAGE and IgG1 immunoblotting of the crude antigen of *Echinococcus granulosus* cyst fluid (EgCF). Crude antigens of EgCF protein extracts were incubated individually with 8 sIgG1 positive sera (*lanes 2-9*), and with disease control (*lane 10*); protein molecular weight marker (*lane 1*). SDS-PAGE, sodium dodecyl sulfate-polyacrylamide gel electrophoresis; sIgG1, specific immunoglobulin G1.

The protein component in the crude antigen of EgCF was analyzed using 1D SDS-PAGE. Several protein bands ranging from 10 to 250 kDa were detected. The most abundant protein band had a molecular weight of 53.5 kDa. The differences in intensity in protein molecular weight of 51.3, 40.5, 27.0, and 24.5 kDa of IgE reaction were detected among individual sera. The 40.5-kDa protein showed positive reaction with 7 sIgE positive sera, and was the major antigen of sIgE in EgCF. The differences in intensity in protein molecular weight of 35.5, 44.6, and 53.2 kDa of IgG1 reaction were detected among individual sera. The 35.5-kDa protein showed positive reaction with 8 sIgG1 positive sera, and was the major antigen of sIgG1 in EgCF. Sera obtained from disease controls showed no reaction against any of the EgCF proteins (Figures 1 and 2).

**Figure 3.**
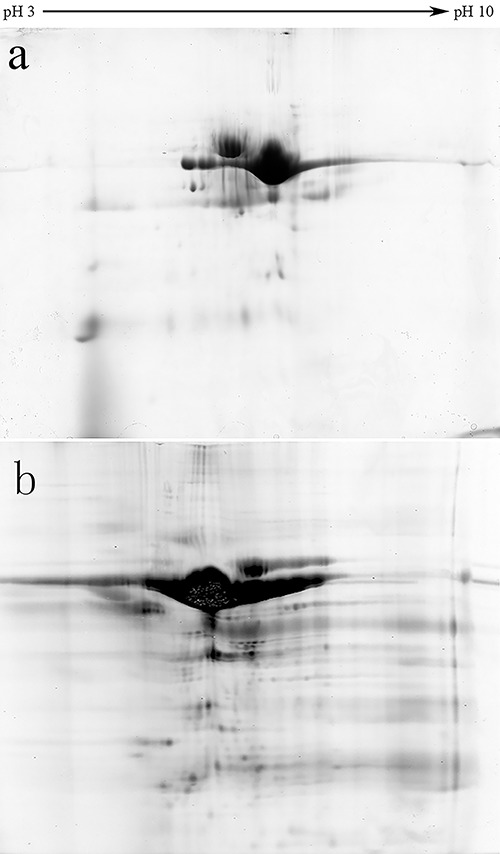
*a*, Seven-centimeter Coomassie blue-stained 2-DE gel of crude antigen of EgCF; *b*, Twenty-four-centimeters fluorescence scanning 2-DE gel of crude antigen of EgCF. 2-DE, two-dimensional gel electrophoresis; EgCF, *Echinococcus granulosus* cyst fluid.

**Figure 4.**
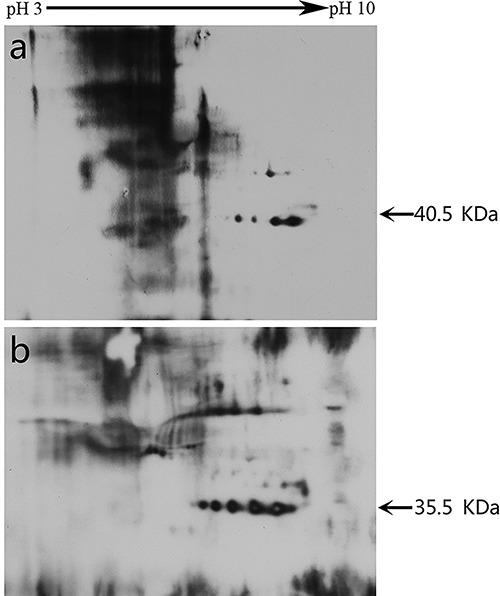
The crude antigen of *Echinococcus granulosus* cyst fluid (EgCF) proteins separated by 2-DE was transferred to the nitrocellulose membrane and probed with sera of patient T1. *a*, IgE immunoblotting was visualized using HRP-labeled mouse anti-human IgE; *b*, IgG1 immunoblotting was visualized using HRP-labeled mouse anti-human IgG1. The sizes of the prominent proteins in kDa are shown on the right-hand side of the figure. 2-DE, two-dimensional gel electrophoresis; IgE, immunoglobulin E; IgG1, immunoglobulin G1.

A representative 7-cm 2-DE gel figure of crude antigen of EgCF stained with Coomassie blue shows the visualization of about 50 protein spots ranging in molecular mass from 10 to 250 kDa, with pI values ranging from 3 to 10. A representative 24-cm 2-DE gel figure of crude antigen of EgCF using laser scanner shows the visualization of about 200 protein spots ranging in molecular mass from 10 to 250 kDa, with pI values ranging from 3 to 10 (Figure 3). To identify the separated proteins, proteins of crude antigen of EgCF were transferred to a nitrocellulose membrane and incubated with T1 patient sera. PI values of four spots ranging from 6.5 to 9.0 comprising 40.5 kDa protein in sIgE immunoblotting and pI values of five spots ranging from 6.5 to 9.0 comprising 35.5 kDa protein in IgG1 immunoblotting showed strong reaction with T1 patient sera (Figure 4).

## Discussion

Anaphylactic shock induced by CE is mediated through sIgE and sIgG1 subclass. In this study, sera from 3 patients with CE who had anaphylactic shock were collected and sIgE- and sIgG1-positive serum were screened using SDS-PAGE, 2-DE, and immunoblotting technique for the corresponding antigen in EgCF. The main antigens of sIgE in EgCF were identified with four protein spots with pI values ranging from 6.5 to 9.0 and a molecular weight of 40.5 kDa. The main antigens of sIgG1 in EgCF were identified with five protein spots with pI values ranging from 6.5 to 9.0 and a molecular weight of 35.5 kDa.

In this study, indirect ELISA method was used to screen sIgE- and sIgG1-positive serum in 20 patients. The absorbance values in controls showed no significant differences than in patients. As plates coated with crude antigen of EgCF were derived from patients with echinococcosis containing anthropogenic IgE and IgG1, it may have produced false-positive results. Hence in the former experiment, EgCF from sheep liver was used to prepare the crude antigen. Seven cases of sIgE-positive serum and 8 cases of sIgG1-positive serum were screened using ELISA. It is interesting to note that 3 cases with anaphylactic shock were caused by spillover of EgCF during the surgery; sIgE and sIgG1 were all positive. The results suggest that the content of serum sIgE and sIgG1 is not only associated with patients with hydatid infections, but also associated with normal allergic components of patients. Results of a preliminary study showed that anaphylactic shock of patients was related to higher content of serum total IgE and IgG1 (11).

IgE immunoblotting results showed that the 40.5-kDa proteins of crude antigen of EgCF had prominent reaction with all seven sIgE-positive sera. In preliminary 1D SDS-PAGE and IgE immunoblotting experiments using DAB, TMB (tetramethylbenzidine), and SuperSignal WestPico did not show obvious immunoblotting bands. However, the use of SuperSignal West Femto Maximum Sensitivity Substrate showed prominent reaction in IgE immunoblotting bands. This suggests that the concentration of sIgE in the serum and sIgE antigen in EgCF was very low. Results of sIgG1 immunoblotting bands also did not distinctly show the corresponding bands or protein point on 1D SDS-PAGE and 2-DE. The results of the study showed that the sIgE and sIgG1 antigen in EgCF belongs to the low abundance proteins. Removing high abundance proteins extraneous to the antigen of cystic fluid protein will concentrate low abundance proteins containing purpose antigen and effectively isolate sIgE and sIgG1 of EgCF antigen.

EgCF antigen for the sensitivity and specificity of specific antibodies was the core index for screening diagnostic antigen. Anaphylactic shock mediated through antibody levels is closely related to the allergic constitution and immune status of the patient; therefore, allergens may not have high sensitivity and specificity of EgCF diagnostic antigens. Studies on sIgE antigen in EgCF are few, with different methods and results (13,14), and the sIgG1 subclass antigen in EgCF has not been reported. AgB is a polymeric lipoprotein with a molecular mass of 120-160 kDa, which under reducing electrophoretic conditions appears to be composed of three subunits with molecular sizes of 8, 16, and 24 kDa. AgB is considered to have high sensitivity and specificity in the diagnosis of echinococcosis (15,16), but it cannot be identified by the sIgE (10). Ag5 is a high molecular mass lipoprotein complex of 67 and 57 kDa antigen in EgCF, which under reducing conditions dissociates into 38 and 22 kDa subunits (13). Results of sIgE and sIgG1 immunoblotting showed no obvious reaction strip in the AgB and Ag5, due to their monomer molecular weight.

### Study limitation and future experimental plans

In this study, valuable serum was collected only from 3 patients with echinococcosis who experienced anaphylactic shock. Whether the recognized antigen could identify the allergen responsible for anaphylactic shock has not yet been validated. Removing high abundance proteins extraneous to the antigen of cystic fluid protein will concentrate low abundance proteins containing target antigen, and effectively isolate sIgE and sIgG1 EgCF antigen. The purified and detected amino acid sequence was effective for the candidate antigens.

In conclusion, sIgE and sIgG1 antigens in EgCF were identified, providing critical insights into cellular and molecular mechanisms underlying anaphylactic shock induced by CE.
